# Dynamics and Extent of Non-Structural Protein 1-Antibody Responses in Tick-Borne Encephalitis Vaccination Breakthroughs and Unvaccinated Patients

**DOI:** 10.3390/v13061007

**Published:** 2021-05-27

**Authors:** Karin Stiasny, Agnes Leitner, Heidemarie Holzmann, Franz X. Heinz

**Affiliations:** Center for Virology, Medical University of Vienna, 1090 Vienna, Austria; agnes.leitner@meduniwien.ac.at (A.L.); heidemarie.holzmann@meduniwien.ac.at (H.H.)

**Keywords:** tick-borne encephalitis, TBE vaccines, vaccination breakthrough, non-structural protein 1, antibody responses

## Abstract

Tick-borne encephalitis (TBE) has a substantial impact on human public health in many parts of Europe and Asia. Effective inactivated purified whole-virus vaccines are in widespread use in TBE-endemic countries. Nevertheless, vaccination breakthroughs (VBTs) with manifest clinical disease do occur, and their specific serodiagnosis was shown to be facilitated by the detection of antibodies to a non-structural protein (NS1) that is produced during virus replication. However, recent data have shown that NS1 is also present in the current inactivated vaccines, with the potential of inducing corresponding antibodies and obscuring a proper interpretation of NS1-antibody assays for diagnosing VBTs. In our study, we quantified anti-virion and anti-NS1 antibody responses after vaccination as well as after natural infection in TBE patients, both without and with a history of previous TBE vaccination (VBTs). We did not find significant levels of NS1-specific antibodies in serum samples from 48 vaccinees with a completed vaccination schedule. In contrast, all TBE patients mounted an anti-NS1 antibody response, irrespective of whether they were vaccinated or not. Neither the dynamics nor the extent of NS1-antibody formation differed significantly between the two cohorts, arguing against substantial NS1-specific priming and an anamnestic NS1-antibody response in VBTs.

## 1. Introduction

Tick-borne encephalitis (TBE) is considered the most important human–pathogenic arthropod-transmitted infectious disease in Europe as well as Central and Eastern Asia, with more than 10,000 cases annually [1]. TBE virus (TBEV) belongs to the family Flaviviridae, genus Flavivirus, comprising a number of other medically relevant viruses such as dengue, Zika, yellow fever (YF), Japanese encephalitis (JE) and West Nile viruses [2]. In Europe, two vaccines are licensed and in widespread use that contain highly purified formalin-inactivated TBEV (European subtype) adsorbed to aluminum hydroxide [3] and confer high protection rates of more than 90% [4]. Nevertheless, clinically manifest vaccination breakthroughs (VBTs) do occur in rare instances [5,6,7,8], and there have been speculations that infection-enhancing phenomena may cause more serious disease in some vaccinated individuals [9]. The antibody pattern found in most VBTs has the characteristics of an anamnestic immune response to the antigens of the virion, resulting in a strong booster of potently neutralizing antibodies, suggesting that immunological priming and memory were not sufficient and/or fast enough to prevent disease in these cases [5,6,7].

During natural flavivirus infections, a set of non-structural proteins is produced that are not part of the viral particle (as used in the vaccines) and play different roles in virus replication [10,11]. Nonstructural protein 1 (NS1) has multiple functions in the viral life cycle, including roles in viral RNA replication as well as virion production [12]. An NS1 dimer is a component of the RNA replication complex and is associated with intracellular membranes as well as the cell surface [12]. NS1 is also secreted from infected cells as a hexamer and circulates in the blood during acute illness [10,11,12]. Assuming that antibodies against NS1 are only formed as a result of virus replication and not vaccination with inactivated vaccines, their detection in serum samples of patients was proposed to allow a clear-cut identification of vaccination breakthroughs (VBTs) [13,14,15]. In addition to its application for serodiagnosis, an NS1 antibody assay can be of value for epidemiological studies, because of its potential to differentiate between the seroprevalence of antibodies due to vaccination or natural infection in regions where part of the population is vaccinated. Recently, however, the potential value of NS1 antibody assays for these purposes was challenged by the demonstration that the two current European TBE vaccines not only contain whole purified inactivated virions but also traces of NS1, which apparently co-purifies and/or remains associated with viral particles during the purification process [16].

Although TBE NS1 antibody assays and analyses have been published [13,14,15,16,17], quantitative data of the levels and kinetics of NS1 IgG antibodies produced in the course of VBTs compared to infections in unvaccinated individuals are not available so far. Such quantitative analyses could also provide clues as to enhancement phenomena and potential differences of the extent of virus replication in these patient groups. In our study, we therefore performed a quantitative analysis of the time course of NS1 antibody responses in TBE patients with and without prior vaccination, using an immunoassay based on recombinant TBE NS1. We could not detect anti-NS1 antibodies in post-vaccination sera and did not observe anamnestic NS1 antibody responses in VBTs, in contrast to the strong booster of antibodies against the virion antigens. There was no significant difference between vaccinated and unvaccinated patients, neither with respect to the quantity nor with respect to the time course of NS1 antibodies produced, providing no evidence for enhanced virus replication in VBTs.

## 2. Materials and Methods

### 2.1. Human Serum Samples

Human serum specimens were originally submitted to the diagnostic laboratory of the Center for Virology, Medical University of Vienna, and were tested for the presence of TBE virion-specific antibodies.

Serum samples of confirmed TBE cases with previous TBE vaccinations (VBT) were available from 18 patients: the first samples were obtained upon hospitalization, and one to two follow-up samples were collected within six weeks after the first sample. A matched control group consisted of 18 TBE patients without flavivirus vaccinations, of which a similar number of archived primary and follow-up samples was available in a comparable period (unvaccinated, UNV).

TBE post-vaccination serum samples were available from 42 vaccinees with a complete schedule of vaccination and positive in the TBE virion IgG ELISA. Sera were sampled from 4 weeks (*n* = 12) to 19 years (*n* = 21) after the last vaccination (median 5 years). For nine individuals the time point of the last TBE vaccination was unknown.

Six samples from individuals with threefold vaccination against TBE, JE and YF were available: TBE (median 3.2 years after vaccination, range 0.3–5.1 years), JE (median 2.1 months after vaccination, range 0.9–4.7 months), and YF (median 4.4 months, range 1.8–5.7 months) vaccination were available.

As negative controls, 27 flavivirus-negative samples (no history of flavivirus vaccinations) from a previous diagnostic study were included [18]. They were tested negative with respect to TBE virion IgG and TBEV neutralizing antibodies. 

### 2.2. Generation of Recombinant TBE NS1

The NS1 protein of TBEV strain Neudoerfl (amino acids NS1 1-352, GenBank accession number U27495) containing a strep-tag (IBA Lifesciences, Göttingen, Germany) was produced in Drosophila Schneider S2 cells as described previously for the flavivirus envelope protein [19]. Briefly, S2 cells stably expressing NS1 were generated by co-transfection with a blasticidin selection vector. Seven to eleven days after induction of expression by CuSO4, the proteins were purified by Streptactin-affinity chromatography following the manufacturer’s protocol (IBA Lifesciences, Göttingen, Germany). Purified NS1 was quantified with the Pierce™ BCA Protein Assay Kit (BCA, bicinchoninic acid) (Thermo Fisher Scientific, Waltham, MA, USA), according to manufacturer’s instructions. Purity was verified with 12% sodium dodecylsulfate-polyacrylamide gel electrophoresis (SDS-PAGE) according to Laemmli [20] (Figure S1A). Validation of the NS1 protein as ELISA antigen was performed by the use of two purified NS1-specific mouse monoclonal antibodies (mabs). The two mabs (5D9, 6E11) were shown to recognize NS1, but not E and prM proteins by immunoprecipitation experiments [21].

### 2.3. Virus Growth, Purification and Inactivation

TBEV strain Neudoerfl was grown in primary chicken cells, harvested 48 h after infection, and purified by 2 cycles of sucrose density gradient centrifugation as previously described [22]. Virus was inactivated with formalin (final dilution 1:2000) for 24 h at 37 °C after purification [23].

### 2.4. NS1 IgG ELISA

Non-treated microtiter plates were coated with 25 ng NS1/well. After incubation of the serum samples, biotin-labeled goat anti-human IgG (Thermo Fisher Scientific) together with Streptavidin−Peroxidase (Sigma, St. Louis, MO, USA) was used for detection. NS1-specific antibodies were quantified in IgG units with a standard polyclonal human post-infection anti-TBEV serum, arbitrarily set at 1000 units (arbitrary units, AU). Standard curves (two-fold dilutions, 7 data points) were fitted using a four-parameter logistic regression (GraphPad Prism 8). The cut-off was based on the validation of the assay with 27 flavivirus-negative sera. NS1 IgG ELISA values <165 AU (mean +3 standard deviations) were defined as negative, 165–200 AU as equivocal, and ≥200 AU (mean +4 standard deviations) as positive. At least two independent experiments were performed for each serum to calculate mean concentrations.

### 2.5. Virion IgG ELISA

TBEV-specific IgG antibodies were analyzed with a 3-layer ELISA using inactivated purified virus coated to the solid phase, as previously described [5,18,24]. Antibodies were quantified in arbitrary IgG units with a standard polyclonal human post-infection anti-TBEV serum set at 1000 units. Standard curves (two-fold dilutions, 7 data points) were fitted using a four-parameter logistic regression (GraphPad Prism 8). The cut-off (220 AU) was determined with 90 flavivirus negative sera in a previous study [18]. At least two independent experiments were performed for each serum to calculate mean concentrations.

In order to harmonize the arbitrary units obtained in the NS1 and virion ELISAs, we adjusted the units obtained by the factor required to shift the standard curves of the NS1 ELISA in such a way that their linear ranges coincided with that of the virion ELISA.

### 2.6. Statistical Analyses

Logarithmic transformation of the data was carried out to obtain approximate normal distribution of antibody concentrations. Student *t*-tests (2 groups) or ANOVA (3 groups) with appropriate post hoc tests (stated in the text) were applied to the transformed data for significance testing. *p* values < 0.05 were regarded as statistically significant.

Statistical analyses were performed with GraphPad Prism 8.

## 3. Results

### 3.1. NS1 IgG Antibodies in TBE Patients and Vaccinees

**Figure 1:**
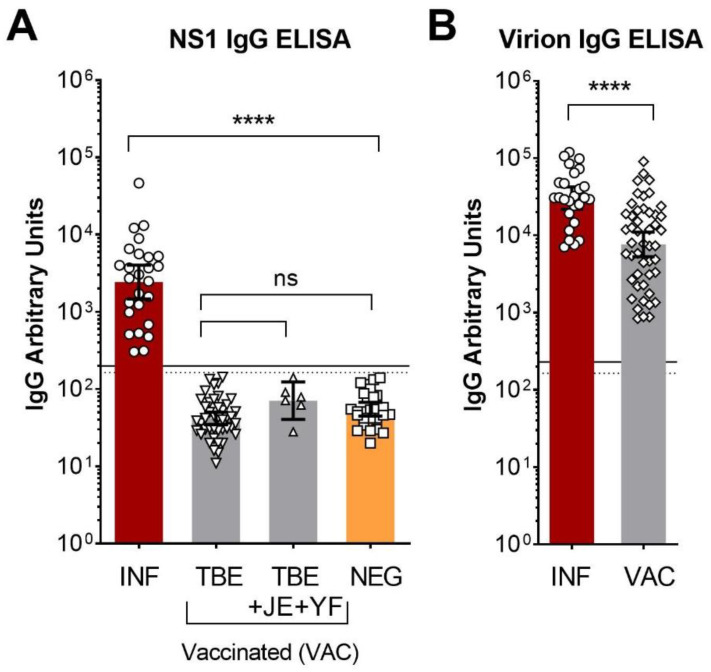
TBE NS1 and virion IgG ELISA with serum samples from flavivirus-negative individuals as well as TBE vaccinees and TBE patients. (**A**) Quantitative analysis of NS1-specific IgG antibodies of human serum samples after TBEV infection without a history of vaccination (INF, *n* = 26), after TBE vaccination (TBE, *n* = 42), after TBE, JE and YF vaccination (TBE + JE + YF, *n* = 6), and negative in TBE NTs (NEG, *n* = 27). (**B**) TBE virion IgG ELISA with all samples from TBE patients (INF, *n* = 26) and TBE vaccinees (VAC, *n* = 48). Data represent geometric means with 95% confidence intervals. Significance was determined with (**A**) ANOVA with Dunnett’s multiple comparisons test or (**B**) *t* test. ns, not significant; ****, *p* < 0.0001. Cut-off positive: solid line, cut-off equivocal: dashed line. TBE, tick-borne encephalitis; JE, Japanese encephalitis, YF, yellow fever; INF, infected, VAC, vaccinated; NEG, negative.

We first quantified NS1-specific IgG antibodies in 26 serum samples collected from unvaccinated TBE patients ~1 to 6 weeks after hospitalization as well as samples from a cohort of 42 individuals with a complete schedule of TBE vaccination (at least three vaccinations), but no history of TBEV infection. We also tested additional six samples from persons, who were not only vaccinated against TBE but also against Japanese encephalitis and yellow fever. As can be seen in Figure 1A, all post-infection sera were NS1-antibody positive, whereas all of the post-vaccination sera yielded a result below the cut-off. As expected, all post-infection and post-vaccination sera had high antibody titers in a virion IgG ELISA (Figure 1B). These data show the validity of the assay for detecting NS1-specific antibodies in post-infection sera, but do not provide direct evidence for NS1-antibody induction through vaccination.

### 3.2. Comparison of Anti-NS1 IgG Responses in TBE Patients with and without Prior Vaccination

To assess possible differences in the extent and dynamics of antibody formation, we analyzed virion- and NS1-specific antibodies in two cohorts of TBE patients of whom consecutive serum samples were available, from the time of hospitalization to release. Cohort A comprised unvaccinated patients (UNV), and cohort B patients with a history of vaccination, referred to as vaccination breakthroughs (VBTs). The characteristics of these cohorts are shown in Table 1, and the collection times of the samples analyzed are displayed in Table 2. This part of the study thus allows a comparison of the two patient groups with respect to the extent and time course of antibody development during hospitalization, and should contribute to answering the following questions: (i) is there evidence for an anamnestic response to NS1 in the VBTs, although NS1-specific antibodies could not be detected in vaccinees without infection? (Figure 1A) (ii) Are there quantitative and/or kinetic differences in the NS1 responses between VBTs and unvaccinated patients that could be indicative of enhanced virus replication in VBTs? For statistical comparisons, we stratified the follow-up samples into two groups: one group contained samples collected 5 to 19 days after the first samples and the second group samples collected 20 to 45 days after the first samples (Table 2). When two samples of one patient fell into the same time interval, only the later one was taken for the calculations of the means.

**Figure 2:**
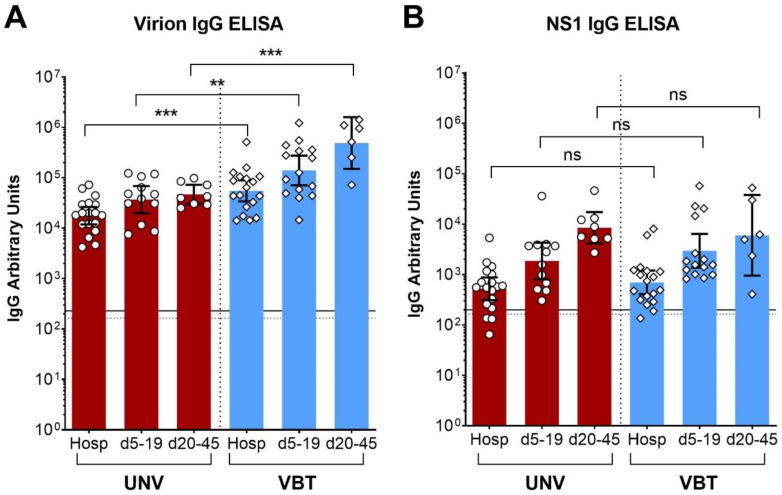
TBE virion- and NS1-specific IgG antibody responses of unvaccinated (UNV) and vaccination breakthrough (VBT) TBE patients in ELISA. (**A**) Virion-specific and (**B**) NS1-specific IgG antibody responses of TBE patients with and without prior vaccination, each group comprising 18 patients. Hosp represents samples obtained upon hospitalization. d5–19 and d20–45 are follow-up samples obtained 5 to 19 days and 20 to 45 days after the first serum, respectively. Data represent geometric means with 95% confidence intervals. The significance of changes in antibody concentrations between the two groups in the different time windows was determined by *t*-tests. ns, not significant; **, *p* < 0.01; ***, *p* < 0.001. Cut-off positive: solid line, cut-off equivocal: dashed line. Hosp, hospitalization; UNV, unvaccinated; VBT, vaccination breakthroughs.

As expected from previous studies [5,6,7], a strong booster occurred in VBTs with respect to the virion-specific antibody response (Figure 2A). At all three time intervals, antibody concentrations were significantly higher in vaccinated than in unvaccinated patients, indicating an anamnestic response in VBTs (Figure 2A). In contrast, no significant differences were found in NS1 antibody responses (Figure 2B), consistent with a primary NS1-antibody response in both groups of patients. A few serum samples of the VBT and UNV groups were NS1 IgG negative upon hospitalization (one negative, one equivocal in the VBT group; three negative in the UNV group) (Figure 2B), but all follow-up samples became positive in the course of hospitalization (Figure 2B). In both groups, the increase in the mean TBE virion- as well as NS1-specific IgG antibody responses during the observation period was significant for the three time windows displayed in the figure (Table 3, ANOVA with Dunnett’s multiple comparisons test, *p* values < 0.05).

## 4. Discussion

The detection of NS1-specific antibodies was proposed to represent a reliable indicator of past TBEV infection, allowing differentiation from antibodies induced by inactivated vaccines and also facilitating diagnosis of VBTs [13,14,15,17]. These studies and conclusions were based on the assumption that the vaccines currently used in Europe (Encepur^®^ and FSME-Immun^®^) do not contain NS1. Recently, a new twist was introduced into the field by the detection of NS1 in both vaccines in current use, either directly by mass spectrometry (Encepur^®^) or indirectly by NS1-antibody induction in vaccine-immunized mice (FSME-Immun^®^) [16]. In our study, none of the samples from TBE-vaccinated individuals (sampled from 4 weeks to 19 years after vaccination) yielded a result above the cut-off in the NS1 IgG ELISA (Figure 1A), suggesting that a potential NS1 response in vaccinees was low and/or rare. Such a conclusion would be consistent with the study conducted by Girl et al. [14], who did not find a single NS1 antibody-positive sample in 49 post-vaccination sera, and with that of Albinsson et al., 2018, who showed a very low anti-NS1 response in only 3 out of 150 post-vaccination sera [17]. According to the authors, TBEV infection during the vaccination period could not be excluded in these cases. In this context, there appears to be some discrepancy with respect to data reported by Salat et al., who found NS1-specific antibodies (albeit at low levels in most instances) in post-vaccination sera using an immunoblot assay [16], although the authors could not completely rule out asymptomatic previous TBEV infections. Possible explanations for observed differences are heterogeneities in the cohorts analyzed with respect to the type of vaccine used, vaccination histories including number and intervals of vaccinations, as well as age distributions of vaccinees. Furthermore, differences in the cut-offs established for the assays used in the different studies and their sensitivities could introduce some variation at the lower part of the scales used for quantification.

We were able to extend previous studies on TBE NS1 antibodies [14,15,17], because we had available consecutive samples taken during the course of disease from both VBTs and TBE patients without vaccination. These samples allowed us to perform a quantitative comparison of the extents and time course of antibody production in TBE patients and VBTs. Consistent with previous results [5], VBTs exhibited a strong anamnestic response against the virion antigens, resulting in antibody titers far exceeding those of TBE patients without previous vaccination (Figure 2A). Evidence for such a significant booster reaction, however, was not obtained with respect to NS1 antibodies (Figure 2B), arguing against substantial priming by NS1 in our cohort of clinically overt VBTs. Theoretically, a solid NS1 response due to its presence in the vaccine could even contribute to protection, as suggested by Salat et al. [16]. The presence or absence of NS1 antibodies could theoretically be a discernable trait between vaccinated individuals with clinically overt or inapparent TBEV infections. Unfortunately, identification of the latter group is quite elusive because diagnostic tests are only performed upon manifest disease.

It is an important result of our study, that neither the time course nor the quantity of NS1-specific antibodies produced differed significantly between TBEV infections in unvaccinated or previously vaccinated individuals, consistent with a primary response in both patient groups (Figure 2B). Since the extent of antibody production might be related to the extent of virus replication, our data do not provide evidence for “vaccine-induced enhancement” of infection in TBE VBTs. This phenomenon has been discussed in relation to disease severity in TBE patients with prior vaccination [9]. Although antibody-dependent enhancement of infection has been linked to more severe disease in sequential dengue virus infections and also following vaccination [25], the discussion whether TBE VBTs might suffer from more severe disease than unvaccinated individuals is still controversial [6,7,8,9,26,27,28]. Detailed comparative and unbiased analyses of the clinical outcomes in the two groups of patients will be necessary to resolve this issue.

In conclusion, the results obtained in our study cohorts argue against a substantial priming of NS1 antibody responses by vaccination, supported by the lack of detectable NS1 antibodies in post-vaccination sera as well as by the absence of a demonstrable anamnestic response in VBTs. However, several variables could be responsible for partially discrepant results in the literature. These include incomplete information on the type of vaccine used in the different studies, differences in the vaccination histories and age of vaccinated individuals, as well as batch-to-batch variation that could result in different amounts of NS1 together with inactivated virus particles in the vaccines. Future studies could address these points, and the direct or indirect quantification of NS1 in inactivated vaccines might be considered as an extension of the current procedures for quality control of vaccine production.

## Figures and Tables

**Table 1:** Characteristics of TBE patients with and without prior vaccination.

TBE Patients	No. of Cases	Age (Years)		Sex (f/m)
		Median	Range	
Unvaccinated	18	57	6–74	8/10
Vaccination breakthroughs	18 (4 ^a^, 14 ^b^)	52	5–75	6/12

^a^ Regular vaccination schedule, ^b^ irregular vaccination schedule.

**Table 2:** Time intervals between first and one or two follow-up serum samples from TBE patients with and without prior vaccination.

TBE Patients		Days between Serum Samples	
	*n*	Median (Days)	Range (Days)
		1st to 2nd samples (d5–19)	
Unvaccinated	12	14	6–17
Vaccination breakthroughs	15	12	5–18
		1st to 3rd samples (d20–45)	
Unvaccinated	8	25	20–45
Vaccination breakthroughs	6	28	21–40

**Table 3:** Statistical evaluation of the increase in the mean TBE virion- and NS1-specific IgG antibody responses at different time points after hospitalization (Figure 2).

**TBE Patients**	**Virion IgG ELISA**				
	**Arbitrary Units (Geometric Mean)**			***p* Values ANOVA (Dunnett’s MCT ^a^)**	
	Hosp ^b^	d5–19	d20–45	Hosp vs. d5–19	Hosp vs. d20–45
Unvaccinated	17,705	36,844	46,866	0.041	0.016
Vaccination breakthroughs	55,105	139,477	490,156	0.039	0.0003
**TBE Patients**	**NS1 IgG ELISA**				
	**Arbitrary units (geometric mean)**			***p* values ANOVA (Dunnett’s MCT ^a^)**	
	Hosp	d5–19	d20–45	Hosp vs. d5–19	Hosp vs. d20–45
Unvaccinated	523	1860	8485	0.008	<0.0001
Vaccination breakthroughs	700	2954	6008	0.007	0.003

^a^ MCT, multiple comparisons test. ^b^ Hosp represents samples obtained upon hospitalization. d5–19 and d20–45 are follow-up samples obtained 5 to 19 days and 20 to 45 days after the first serum, respectively.

## Data Availability

All data are contained within the article and the Supplementary Materials.

## Supplementary Materials

The following are available online at https://www.mdpi.com/article/10.3390/v13061007/s1, Figure S1: Characterization of the TBE NS1 protein.
