# Supplementary material for: Dynamics and Extent of Non-Structural Protein 1-Antibody Responses in Tick-Borne Encephalitis Vaccination Breakthroughs and Unvaccinated Patients
Source: Viruses. 2021 May 27;13(6):1007. doi: 10.3390/v13061007 (PMC8228328; doi:10.3390/v13061007)
Supplement: Supplementary file 1 [file viruses-13-01007-s001.zip › viruses-1229762-supplementary.pdf]

Figure S1: Characterization of the TBE NS1 protein

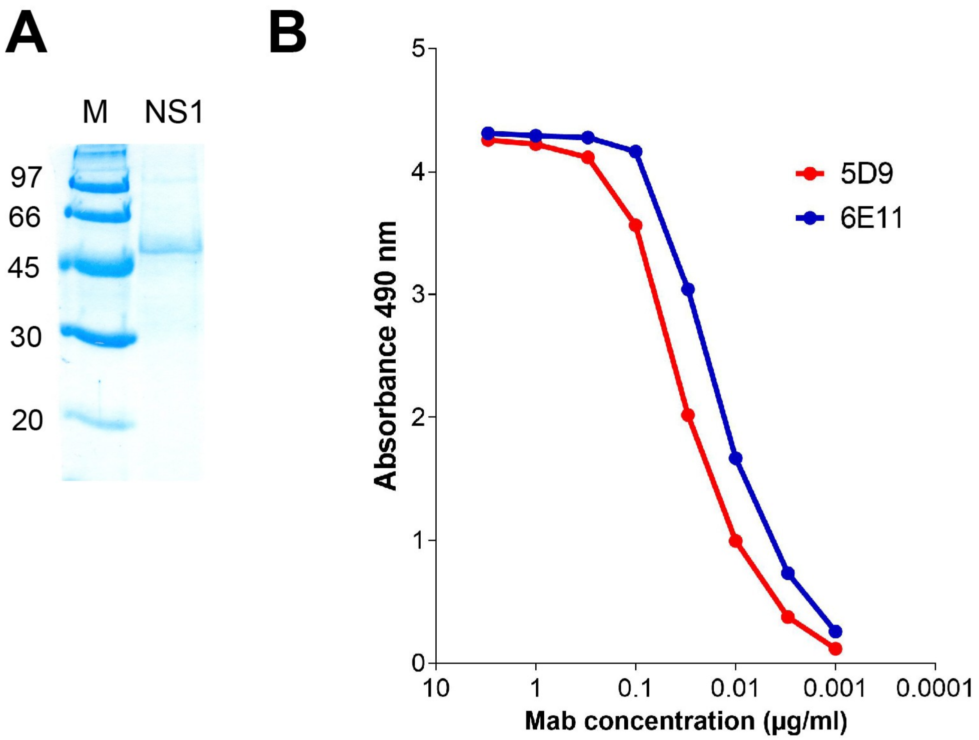

(A) SDS-PAGE of purified NS1 protein. M, marker. The molecular weights (kD) of the marker proteins are indicated on the left.
(B) ELISA with two mouse monoclonal antibodies (5D9, 6E11) recognizing TBE NS1. NS1 was coated to the solid phase, and titrations of purifed antibodies were used for detection together with rabbit-anti-mouse IgG horseradish peroxidase. Mab, monoclonal antibody.
